# From medical to pedagogical care: A bibliometric review of AI agent research in education

**DOI:** 10.1097/MD.0000000000050895

**Published:** 2026-10-09

**Authors:** Xinhuang Tang, Shukun Chen, Jinyan Chen

**Affiliations:** a School of Foreign Languages and Culture, Guangdong University of Finance, Guangzhou, Guangdong, China; b School of Foreign Languages, Guangzhou Huashang College, Guangzhou, Guangdong, China.

**Keywords:** AI agents, bibliometric analysis, ethical AI, medical care

## Abstract

**Background:**

The integration of artificial intelligence (AI) agents, including chatbots and virtual tutors, into education has expanded rapidly; however, systematic quantitative analysis of research trends in this field remains limited. Notably, the intellectual foundations of AI agent research lie not in education but in medicine, where AI agents were first extensively deployed and studied in clinical, experimental, and pedagogical contexts.

**Methods:**

In this study, a bibliometric analysis of 377 records retrieved from the Web of Science was conducted using VOSviewer to map the evolution, thematic focus, and collaborative networks within this field.

**Results:**

The study showed AI agent research originated strongly in healthcare, with educational applications emerging more recently. Key research themes encompassed AI technologies, human–computer interaction, pedagogical applications, and ethical considerations. Collaborative efforts were led by the USA and China, supported by active European and Asian research hubs. The findings revealed a dual narrative of significant benefits for personalized learning and learner well-being alongside persistent challenges such as algorithmic bias and accessibility gaps.

**Conclusion:**

To realize the transformative potential of AI in education, future research should prioritize the development of adaptive algorithms, robust ethical frameworks, inclusive design, and interdisciplinary collaboration. By focusing on these areas, the field can work toward overcoming technical, ethical, and logistical barriers, thereby ensuring that AI acts as a sustainable force for equity and excellence in education.

## 1. Introduction

The rapid advancement of artificial intelligence (AI) has propelled the integration of AI agents such as chatbots, virtual tutors, and conversational systems into diverse sectors into education. These agents are increasingly being recognized for their potential to transform traditional learning environments by offering personalized, interactive, and scalable support.^[1]^ In educational contexts, AI agents demonstrate significant importance in enhancing student engagement, providing real-time feedback, and fostering social connections. For instance, chatbots in higher education can assist learners in acquiring foundational knowledge through interactive and confidential interactions,^[2]^ while AI-driven reading companions in primary schools improve literacy by simulating social partnerships that sustain interest and participation.^[3]^ Moreover, studies have highlighted the role of AI agents in facilitating collaborative learning, such as enabling students to refine their skills through AI-guided feedback in dance education^[4]^ or improving psychological well-being via social robot interventions.^[5]^ The key characteristics of these agents include adaptability to individual learning needs, the ability to automate administrative tasks, and the capacity to support affective domains such as motivation and self-regulation.^[6,7]^

Critically, the intellectual and empirical foundations of AI agent research lie not in education but in healthcare. As revealed in the citation analysis (Section 3.1), the earliest and most prolific applications of AI agents, including chatbots, virtual tutors, and conversational systems, emerged in clinical, experimental, and pedagogical contexts within medicine. Documents from our analysis demonstrate that AI agents have been extensively deployed for chronic disease management,^[8,9]^ patient education in oncology,^[10,11]^ sexual and reproductive health advice,^[12,13]^ and clinical laboratory medicine.^[14]^ These healthcare applications have generated a dense corpus of empirical studies, design heuristics, and failure analyses, such as rigorous evaluations of user acceptability, safety, effectiveness, and ethical considerations, that now function as the actual knowledge base for agent-based scholarship across all domains.

Previous review studies have contributed to an understanding of the scope and challenges of AI in education. For example, Casheekar et al provided a comprehensive analysis of AI-powered conversational agents (CAs), such as ChatGPT, examining their technical backgrounds, applications, and ethical issues.^[15]^ Similarly, Dwivedi et al synthesized the opportunities and impacts of generative AI across education and other fields, emphasizing personalized learning and ethical considerations. Følstad et al outlined a research agenda for chatbot development focusing on user experience and interdisciplinary collaboration.^[16]^ However, these reviews primarily adopt qualitative or narrative approaches and often lack systematic quantification of research trends, patterns, and networks within the field. Despite the growing literature, there remains a scarcity of bibliometric methods to map the evolution, thematic clusters, and collaboration networks of AI agent applications in education. Such an approach would offer a data-driven perspective on research productivity, emerging focus, and knowledge structures. Therefore, this study aimed to address the following research questions through a bibliometric method:

1. What are the foundational and influential publications that have shaped the field of AI agents research in educational contexts?
2. What are the predominant research topics and thematic clusters within the domain of AI agents in education?
3. How is the research landscape structured in terms of collaborative networks and contributing disciplines?
4. What are the emerging trends and potential future research directions suggested by the current literature?

The first question aims to identify key authors, seminal works, and intellectual foundations of the field through citation analysis. The second question seeks to map the conceptual structure of the field, identify major research fronts, and determine how they have evolved over time using a co-occurrence analysis. The third question investigates disciplinary structure through a bibliographic coupling analysis of journals. The last question focuses on identifying frontier topics and research gaps by content analysis of key works identified through bibliometric analysis.

## 2. Materials and methods

To achieve the above-mentioned objectives, this study adopts a bibliometric approach to review studies concerning AI agents and education using the VOSviewer tool. VOSviewer is a software tool used for constructing and visualizing bibliometric networks. It is widely used in scientific mapping and knowledge domain analyses. The software can create network visualizations such as coauthorship, co-citation, and keyword co-occurrence networks. VOSviewer supports data from sources such as Scopus and Web of Science (WoS) and allows users to explore relationships between items through interactive maps. Its user-friendly interface and powerful visualization capabilities make it popular for research evaluation and trend analysis.

The bibliometric analysis conducted in this study is based on the large-scale academic database of the WoS, specifically the Science Citation Index Expanded and the Social Sciences Citation Index, which were imported into VOSviewer.

The analytical dimensions of this study focus on several key bibliometric indicators: citation-document analysis (presenting a structured map of scholarly literature on “AI agent” and “education”), co-occurrence-all keywords analysis (examining the distribution and prevalence of specific terminologies within the citation contexts of AI agents and education), bibliographic coupling-document analysis (identifying thematic similarities between scholarly works that refer to common third-party sources), and bibliographic coupling-countries analysis (depicting the scholarly contributions of the 25 countries and regions to AI agent-based education). Table 1 lists the parameters set in VOSviewer for each analytical dimension mentioned above. These multifaceted analytical dimensions collectively enable a comprehensive examination of the research structure and knowledge diffusion patterns within the field.

**Table 1:** Analytical dimensions and their related parameters set in VOSviewer.

Dimensions	Type of analysis	Unit of analysis	Counting method	Minimal number set	Numbers selected	Numbers of cluster
Citation-document analysis	Citation	Documents	–	10	97	5
Co-occurrence-all keywords analysis	Co-occurrence	All key words	Full counting	5	68	6
Bibliographic coupling-documents analysis	Bibliographic coupling	Documents	Full counting	20	51	9
Bibliographic coupling-sources analysis	Bibliographic coupling	Sources	Full counting	5	8	2
Bibliographic coupling-countries analysis	Bibliographic coupling	Countries	Full counting	5	25	6

The dataset comprises 377 scholarly records retrieved from the WoS Core Collection (the Science Citation Index and the Social Sciences Citation Index) using the topic search “AI agent” AND “education.” Table 2 presents the basic descriptive characteristics of this dataset.

**Table 2:** Descriptive statistics of the retrieved dataset (N = 377).

Characteristics	Details
Publication years	2015–2025
Document types	Article (289), Early Access (48), Proceedings paper (32), Review (8)
Top 5 source titles	Interactive Learning Environments (19), Education and Information Technologies (16), Computers & Education (14), IEEE Access (11), Sustainability (9)
Top 5 WoS categories	Education & Educational Research (142), Computer Science Information Systems (89), Computer Science Interdisciplinary Applications (67), Engineering Electrical & Electronic (34), Health Care Sciences Services (28)

WoS = Web of Science.

This descriptive overview establishes the basic corpus characteristics before proceeding to network-based analyses.

Meanwhile, this study is a bibliometric analysis based solely on publicly available scholarly literature retrieved from the WoS Core Collection. It did not involve human participants, animals, clinical trials, or any form of primary data collection from individuals. Therefore, ethical approval from an institutional review board or ethics committee was not required, and informed consent was not applicable. All analyses were conducted on published documents, and no personal or sensitive information was accessed or processed.

## 3. Bibliometric findings

This section reports the bibliometric results generated through 5 analytical dimensions: citation-document analysis, co-occurrence all keywords analysis, bibliographic coupling-document analysis, bibliographic coupling-source analysis, and bibliographic coupling-country analysis.

### 3.1. Citation-document analysis

Figure 1 is mapped based on the citation-document analysis. The network visualization presents a structured map of scholarly literature on “AI agent” and “education, clustered into 5 distinct color-coded groups (clusters 1–5) based on thematic similarity and citation relationships. Each cluster represents a unique research focus interconnected through shared methodologies, technologies, or application domains. Below is a synthesis of the key research topics, a description of the network structure, and an analysis of the inter-cluster relationships.

**Figure 1.**
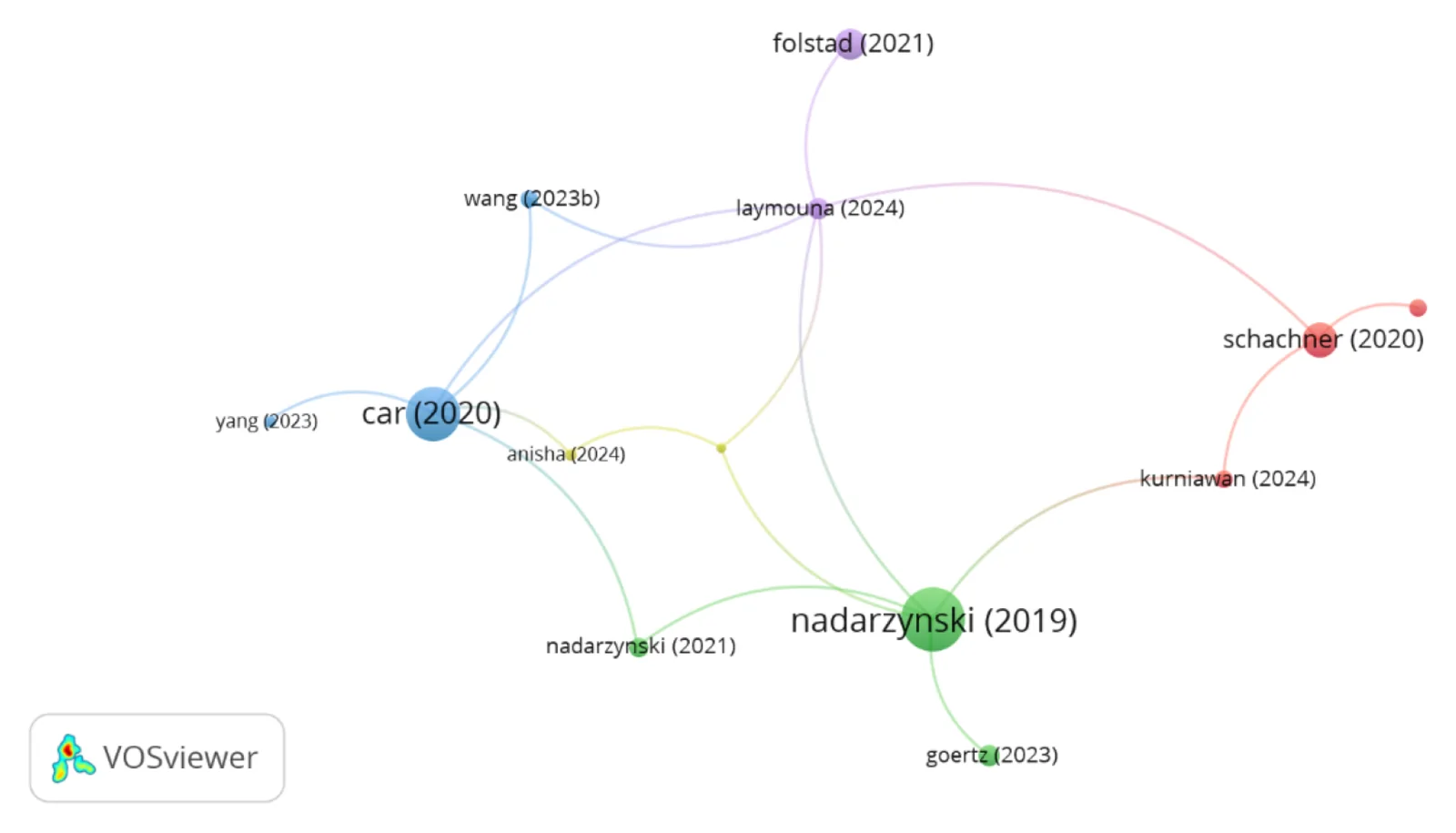
Citation-document analysis based on VOSviewer network visualization.

#### 3.1.1. Cluster 1 (blue color): AI-augmented training systems in specialized contexts

This cluster explores the integration of AI into immersive training environments, with a focus on improving outcomes in high-stakes and skill-dependent sectors. A standout study developed an AI-driven virtual reality system (e.g., iSafeCom) for migrant construction workers, combining ChatGPT-powered real-time instruction with simulated safety scenarios. By addressing language barriers and trainer limitations, the system enhances knowledge retention (23% score improvement) across diverse educational backgrounds.^[17]^ Complementary research reviews AI-powered chatbots for chronic disease management, emphasizing their role in self-management support and highlighting gaps in evidence rigor. Another study systematically examined AI dialogue agents for chronic conditions, identifying their functions (e.g., data collection and diagnosis) but noting inconsistencies in AI architecture reporting. Overall, cluster 1 forms the foundation for practical applications of AI in specialized training (construction safety) and healthcare, showing how intelligent agents can personalize and contextualize learning or support.

#### 3.1.2. Cluster 2 (green color): patient-centered AI chatbots in healthcare delivery

Centered on AI chatbots as tools for direct patient engagement, this cluster investigates their acceptability, usability, and impact across diverse medical contexts. One study evaluates a prostate cancer education chatbot (PROSCA) and demonstrated high user satisfaction (89% knowledge gain) and willingness to adopt such tools clinically. A mixed-methods study assessed public attitudes toward AI-led health chatbots, revealing 67% acceptance linked to perceived utility but highlighting barriers such as low information technology (IT) literacy. Another qualitative analysis identified facilitators (anonymity and convenience) and barriers (privacy concerns and lack of empathy) for AI chatbots in sexual/reproductive health advice. Cluster 2 bridges AI technology with patient-centered care, emphasizing usability, trust, and ethical considerations in chatbot designs for health education and support.

#### 3.1.3. Cluster 3 (orange color): AI dialogue agents in clinical and oncological settings

Focusing on clinical environments, this cluster examines the roles of AI dialogue agents in oncology and laboratory medicine. A scoping review critiques the inconsistent evaluation of healthcare dialogue agents, noting their prevalent use in treatment monitoring and patient education but lacking standardized assessments. A narrative review highlights AI chatbots’ effectiveness in oncology (e.g., breast/ovarian cancer) for improving patient-provider communication, though it calls for more user testing. Another study explored AI chatbots in clinical laboratory medicine, indicating their potential to explain test results but underscoring reliability challenges. Cluster 3 links AI’s technical capabilities (e.g., natural language processing) to critical care domains, stressing the need for rigorous validation in high-stakes medical contexts.

#### 3.1.4. Cluster 4 (purple color): AI for chronic disease and global health equity

To address global health disparities, this cluster investigates AI-driven CAs to manage noncommunicable diseases and rare conditions in low-resource settings. A narrative synthesis of 43 studies revealed high acceptance of anthropomorphic CAs for NCD self-management (e.g., nondrug interventions) but noted gaps in personalized, ethical design.^[18]^ A case study developed a multilingual AI chatbot (Saytu Hemophilie) for Senegalese hemophilia patients, achieving superior usability and cultural adaptability, with implications for African communities.^[19]^ This cluster expands AI’s role in equitable healthcare delivery by focusing on underserved populations and chronic conditions through culturally tailored, scalable solutions.

#### 3.1.5. Cluster 5 (gray color): foundational reviews and conceptual frameworks

Serving as the conceptual backbone, this cluster synthesizes broader trends and challenges in research on AI agents. A scoping review maps 47 studies on healthcare dialogue agents, identifying dominant applications (e.g., therapy and patient education) and calling for stricter evaluations of acceptability, safety, and effectiveness. A narrative review of oncology chatbots and a technical analysis of lab-medicine AI agents further underscores gaps in standardization and empirical validation. Cluster 5 provides overarching insights into research gaps, methodologies, and future directions by connecting empirical studies to theoretical frameworks.

In general, the citation network revealed 2 crucial insights into the developmental trajectory of AI agent research. First, healthcare emerged as the earliest and most prolific arena of application: long before “AI in education” became a recognizable subfield, clinical settings had already incubated a dense corpus of empirical studies, design heuristics, and failure analyses that now function as the actual knowledge base for agent-based scholarship. Second, strikingly, none of the 5 document clusters foregrounds education as a primary domain; instead, the cited references in educational studies are traceable almost entirely to medical chatbot and dialogue-agent literature. This absence indicates that the systematic investigation of AI agents in education is a relatively new domain. Consequently, the medical field has not merely pioneered practical deployments but has supplied literature and theoretical foundations for research on emerging educational AI agents.

### 3.2. Co-occurrence all keywords analysis

Figure 2 shows the clustering of 68 keywords, among which 6 clusters and 17 links were formed. This analysis examines the distribution and prevalence of specific terminologies within the citation contexts of AI agents and education.

**Figure 2.**
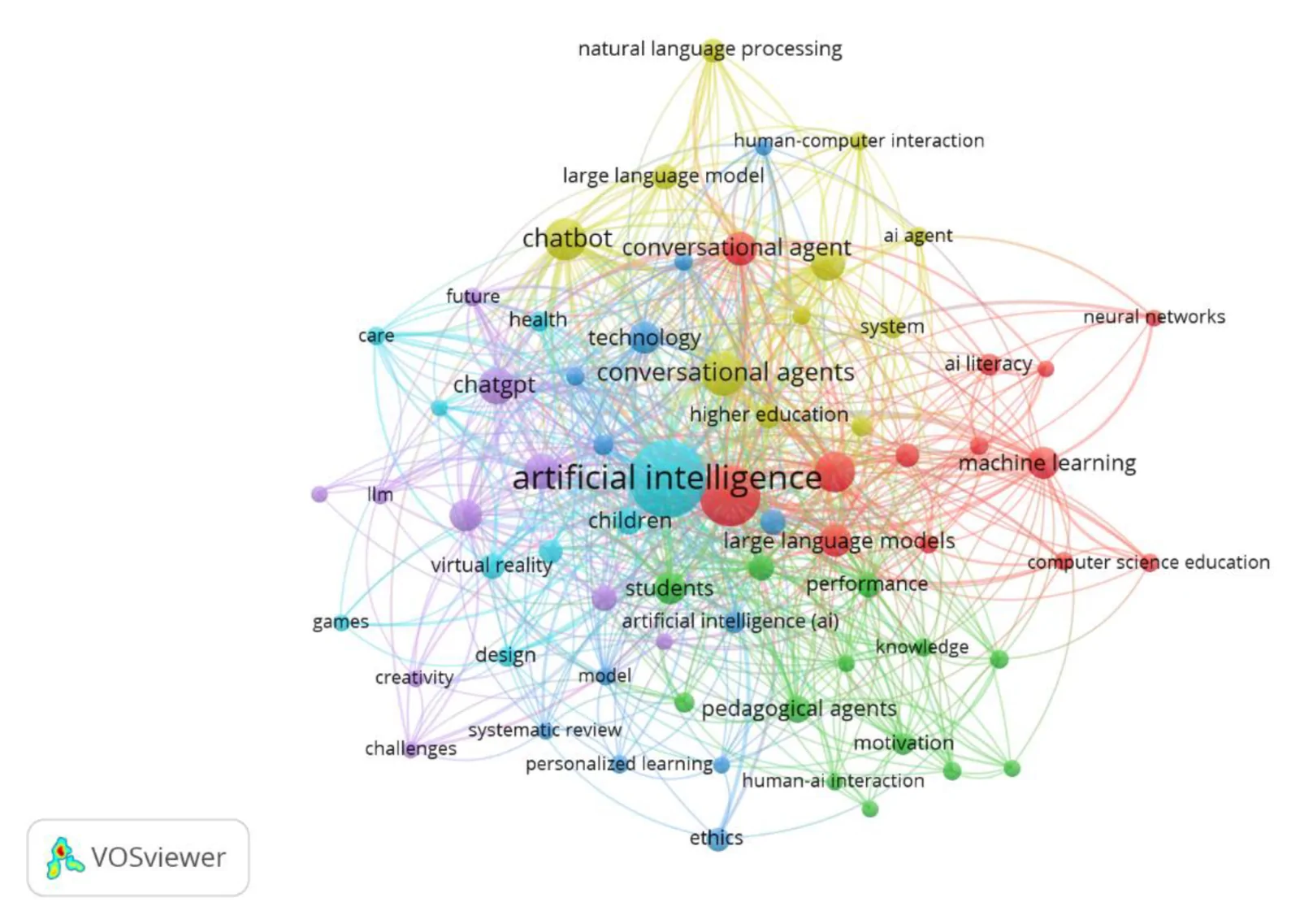
Clustering of keywords in educational AI agents research. AI = artificial intelligence.

#### 3.2.1. Red network: core AI and machine-learning concepts

The dominant themes were “artificial intelligence,” “machine learning,” “neural networks,” “large language models” and “AI agent.” These terms delineate a coherent hot topic: the iterative integration and rapid evolution of AI/ML (machine learning) techniques particularly transformer-based large language models (LLMs) as the principal catalysts for next-generation AI agents. Studies clustered around this network typically pursue performance gains in natural-language understanding and generation, contextual reasoning, and cross-domain adaptability while retaining deep neural architectures as the core computational substrate that enables complex, high-level learning and decision-making.

#### 3.2.2. Yellow network: interaction-related concepts

The yellow network identifies “natural language processing,” “human–computer interaction,” “conversational agents” and “chatbots” as major themes. The corresponding research front centers on refining the sociotechnical interface between humans and machines by elevating the pragmatic quality of language-based interaction. Investigations emphasize gains in semantic accuracy, dialogue coherence, and contextual appropriateness, seeking to transform rule-bound chatbots into adaptive conversational partners that can analyze elliptical or multi-intent queries, track inter-turn contexts, and generate responses perceived as both helpful and relationally natural. This strand thus complements the LLM-centric agenda; whereas the latter supplies increasingly powerful language generation foundations, the former translates these foundations into measurable improvements in user experience, acceptance, and task-specific utility.

#### 3.2.3. Green network: educational and pedagogical applications

The green network signals a distinct yet rapidly blending research domain dedicated to the educational and pedagogical deployment of AI. Keyword frequencies and burst indices converge on “artificial intelligence,” “children,” “students,” “higher education,” “pedagogical agents,” “personalized learning” and “systematic review” framing a hot topic that probes how intelligent systems can be recruited to scaffold instruction across developmental stages. Rather than merely integrating chatbots into classrooms, the emergent literature pursues the theory-driven design of pedagogical agents that adapt content sequencing, feedback granularity, and motivational scaffolding to the cognitive profile of individual learners. Synthesizing evidence through systematic review protocols, scholars are beginning to adjust effect sizes for achievement, engagement, and retention gains while simultaneously exposing implementation gaps – data privacy, algorithmic bias, and teacher empowerment – that condition sustainable uptake. In doing so, the educational strand extends the medical-origin knowledge base documented in earlier clusters, recontextualizing agent architectures from clinical decision support to learner-centered cognition, thereby asserting education as a legitimate, if still junior, arena of AI agent innovation.

#### 3.2.4. Blue network: broader technological and social aspects

The blue network delineates macro-level discourse that examines the entanglement of emerging technologies, particularly AI, with broader social, cultural, and ethical fabrics. Recurrent keywords such as “technology,” “future,” “care,” “health,” “design,” “games” and “creativity” center on a hot topic concerned less with algorithmic specifics than with systemic reverberations. Studies in this field have examined how AI-infused systems reconfigure care pathways in healthcare, catalyze participatory design paradigms, or function as co-creative partners in game-based environments that foster new forms of human imagination. Crucially, scholarship here anticipates forward-looking scenarios: the redistribution of agency between humans and machines, the emergence of post-anthropocentric design ethics, and long-term implications for social well-being, inclusion, and cultural sustainability.

#### 3.2.5. Purple network: challenges and ethical considerations

Purple-network co-word maps foreground “challenges,” “ethics,” “human-AI interaction,” “motivation,” “performance” and “knowledge” as a lexically stable yet semantically expanding cluster. The corresponding hot topic revolves from technical optimization to the critical interrogation of the socio-cognitive frictions that emerge when algorithmic agents intermediate human activity. Empirical work within this stream demonstrates dual phenomena: the motivational crowding-out that can accompany overreliance on adaptive feedback loops and the performance volatility induced by opaque recommendation regimes. Complementing these micro-level findings, conceptual contributions propose integrative ethical frameworks (explicability, fairness, and pedagogical beneficence) tailored to educational and social contexts in which developmental stakes are high.

#### 3.2.6. Teal network: technology-based AI agents’ application in the research domains

The teal network condenses around terms such as “technology,” “future,” “health,” “higher education,” “performance,” “students” and “care.” Centered around “technology,” this cluster links to key nodes like “health” and “higher education,” reflecting the transformative role of AI in healthcare and education systems. The inclusion of “performance” and “students” highlights research on AI’s impact on learning outcomes, while “care” and “future” suggest forward-looking studies on AI’s ethical and practical dimensions in caregiving contexts. This cluster demonstrates how foundational AI technologies intersect with real-world applications that shape human development and well-being. The dense connections between nodes reveal interdisciplinary approaches to evaluating the societal integration of AI.

Based on the co-occurrence-all keywords analysis, studies on AI agents and education seem to evolve through several interconnected domains. The core technological development (Red Network) focuses on advancing fundamental AI concepts, such as neural networks and LLMs, to create more capable and intelligent AI agents. This technical progress directly enables sophisticated human-computer interaction (Yellow Network), where research aims to make CAs more natural and context-aware.

### 3.3. Bibliographic coupling-documents analysis

Bibliographic coupling assumes that 2 documents share a thematic relationship if they cite a common third document. For this analysis, the following parameters are set in VOSviewer: analysis type = bibliographic coupling, unit of analysis = documents, counting method = full counting, minimum number of citations of a document = 20. This threshold yielded 51 documents. The number of clusters (*k* = 9) was determined automatically by VOSviewer using its default resolution parameter (resolution = 1.00), which employs a modularity-based clustering algorithm. This algorithm maximizes intra-cluster similarity while minimizing inter-cluster similarity without manual intervention. No manual adjustment of cluster number was performed to ensure reproducibility. For each resulting cluster, Table 3 lists the studies with the total link strength (>20), a VOSviewer metric which indicates the sum of coupling strengths between a given document and all other documents in the network, thereby identifying the most representative or centrally positioned studies within each thematic cluster.

**Table 3:** Representative references in each cluster (by highest total link strength [>20]).

Clusters	Author	Articles	Journal
Cluster 1	Al-Sharafi et al^[20]^	Understanding the impact of knowledge management factors on the sustainable use of AI-based chatbots for educational purposes using a hybrid SEM-ANN approach	Interactive Learning Environments
	Dwivedi et al^[1]^	So what if ChatGPT wrote it?” Multidisciplinary perspectives on opportunities, challenges and implications of generative conversational AI for research, practice and policy	International Journal of Information Management
	Bilquise et al^[21]^	Investigating student acceptance of an academic advising chatbot in higher education institutions	Education and Information Technologies
Cluster 2	Chen et al^[2]^	Artificial intelligence (AI) student assistants in the classroom: designing chatbots to support student success	Information Systems Frontiers
	Ait Baha et al^[22]^	The impact of educational chatbot on student learning experience	Education and Information Technologies
	Zhai^[23]^	A systematic review on cross-culture, humor and empathy dimensions in conversational chatbots: the case of second language acquisition	Heliyon 8
Cluster 3	Laymouna et al^[24]^	Roles, users, benefits, and limitations of chatbots in health care: rapid review	Journal of Medical Internet Research
	Bian et al^[25]^	Artificial intelligence–assisted system in postoperative follow-up of orthopedic patients: exploratory quantitative and qualitative study	Journal of Medical Internet Research
	Kurniawan et al^[8]^	A systematic review of artificial intelligence powered (AI-powered) chatbot intervention for managing chronic illness	Annals of Medicine
Cluster 4	Casheekar et al^[15]^	A contemporary review on chatbots, AI-powered virtual conversational agents, ChatGPT: applications, open challenges and future research directions	Computer Science Review
	Følstad et al^[16]^	Future directions for chatbot research: an interdisciplinary research agenda	Computing
	Jalil et al^[7]^	ChatGPT and software testing education: promises & perils	2023 IEEE International Conference on Software Testing, Verification and Validation Workshops (ICSTW)
Cluster 5	Van Brummelen et al^[26]^	Alexa, can I program you?”: student perceptions of conversational artificial intelligence before and after programming Alexa	Athens
	Long et al^[27]^	Family learning talk in AI literacy learning activities	CHI ’22
	Ng et al^[28]^	Artificial intelligence (AI) literacy education in secondary schools: a review	Interactive Learning Environments
Cluster 6	Nadarzynski et al^[14]^	Barriers and facilitators to engagement with artificial intelligence (AI)-based chatbots for sexual and reproductive health advice: a qualitative analysis	Sexual Health
	Nadarzynski et al^[12]^	Acceptability of artificial intelligence (AI)-led chatchatbot services in healthcare: a mixed-methods study	Digital Health
	Car et al^[14]^	Conversational agents in health care: scoping review and conceptual analysis	Journal of Medical Internet Research
Cluster 7	Hwang et al^[29]^	How artificial intelligence (AI) supports nursing education: profiling the roles, applications, and trends of AI in nursing education research	Interactive Learning Environments
	Liaw et al^[30]^	Artificial intelligence in virtual reality simulation for interprofessional communication training: mixed method study	Nurse Education Today
	Stamer et al^[31]^	Artificial intelligence supporting the training of communication skills in the education of health care professions: scoping review	Journal of Medical Internet Research
Cluster 8	Perez-Liebana et al^[32]^	General video game AI: a multi-track framework for evaluating agents, games and content	IEEE Transactions on Games
	Ijaz et al^[33]^	Generation algorithms virtual worlds vs books and videos in history education	Interactive Learning Environments
Cluster 9	Jeong et al^[5]^	Deploying a robotic positive psychology coach to improve college students’ psychological well-being	User Modeling and User-Adapted Interaction
	Kang et al^[4]^	Dancing on the inside: a qualitative study on online dance learning with teacher-AI cooperation	Education and Information Technologies

AI = artificial intelligence.

Nine clusters of studies are identified through bibliographic coupling-document analysis, as shown in Figure 3. Table 3 presents information on the representative references within each cluster, defined as those with the highest total link strength (>20). Based on these representative references and their full texts, the following characterization of each cluster’s thematic focus is provided.

**Figure 3.**
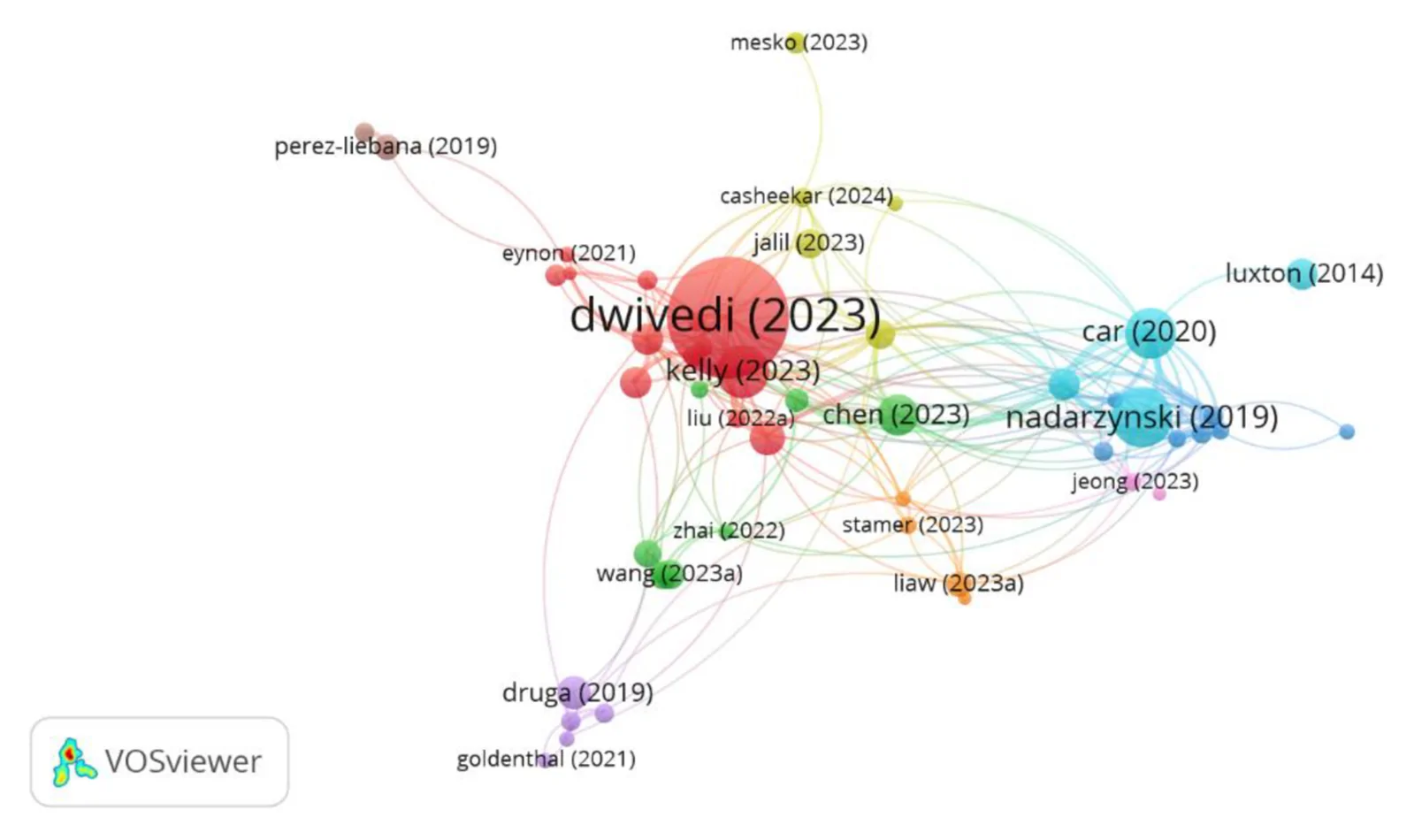
Bibliographic coupling-documents analysis.

Cluster 1 focuses on the application of AI in education, particularly AI chatbots and intelligent tutoring systems. This cluster explores how AI technologies can enhance student learning experiences, improve educational outcomes, and support personalized learning. Key research topics include the design and implementation of AI chatbots, the impact of AI on student engagement and motivation, and ethical considerations surrounding the use of AI in educational settings.

Cluster 2 examines the role of intelligent agents in facilitating communication and collaboration among students and educators. This cluster investigates how intelligent agents can support collaborative learning environments, enhance teacher-student interactions, and provide personalized feedback and support. Key research topics include the development of intelligent tutoring systems, the use of natural language processing to enhance communication, and the impact of intelligent agents on student learning outcomes.

Cluster 3 explored the use of intelligent agents in healthcare education and training. This cluster focuses on how intelligent agents can be used to simulate real-world scenarios, provide hands-on training experience, and enhance the skills and competencies of healthcare professionals. Key research topics include the development of virtual reality simulations, the use of intelligent agents for patient education, and the impact of intelligent agents on healthcare outcomes.

Cluster 4 investigates the potential of intelligent agents in supporting lifelong learning and professional development. This cluster examines how intelligent agents can provide personalized learning experiences, support self-directed learning, and facilitate continuous skill development. Key research topics include the design of intelligent tutoring systems for adult learners, the use of intelligent agents for career guidance, and the impact of intelligent agents on professional development outcomes.

Cluster 5 explores the ethical and social implications of using intelligent agents in education and society. This cluster examines the potential risks and benefits of using intelligent agents, including issues related to privacy, security, bias, and accountability. Key research topics include the ethical design of intelligent agents, the impact of intelligent agents on social norms and values, and the role of intelligent agents in promoting social justice and equity.

Cluster 6 focuses on the technical aspects of developing and implementing intelligent agents in education. This cluster examines the algorithms, architectures, and platforms used to develop intelligent agents, as well as the challenges and opportunities associated with integrating these technologies into educational settings. Key research topics include the development of natural language processing algorithms, the design of intelligent tutoring systems, and the integration of intelligent agents into existing educational technologies.

Cluster 7 examines the impact of intelligent agents on student learning outcomes and academic achievement. This cluster investigates how intelligent agents can support student learning, improve academic performance, and enhance educational equity. Key research topics include the impact of intelligent tutoring systems on student learning outcomes, the use of intelligent agents for personalized learning, and the role of intelligent agents in promoting academic success.

Cluster 8 explores the potential of intelligent agents to support mental health and well-being in educational settings. This cluster examines how intelligent agents can provide emotional support, promote positive mental health behaviors, and enhance overall well-being. Key research topics include the development of intelligent agents for mental health support, the impact of intelligent agents on students’ mental health outcomes, and the role of intelligent agents in promoting positive mental health practices.

Cluster 9 investigates the role of intelligent agents in enhancing teachers’ professional development and instructional practices. This cluster examines how intelligent agents can support teacher learning, improve instructional quality, and enhance teacher-student interactions. Key research topics include the development of intelligent tutoring systems for teachers, the use of intelligent agents for instructional coaching, and the impact of intelligent agents on teacher professional development outcomes.

Network visualization of 9 clusters highlights the diverse and interconnected nature of research in the fields of intelligent agents and education. These 9 clusters collectively examine the multifaceted role of intelligent agents, especially AI chatbots and intelligent tutoring systems, in education, healthcare, and society.

### 3.4. Bibliographic coupling-sources analysis

To identify which disciplines engage with AI agent research in education, a bibliographic-coupling analysis of publication sources is performed. Figure 4 shows a summary of important literature sources in the AI agent-based education field from the analysis.

**Figure 4.**
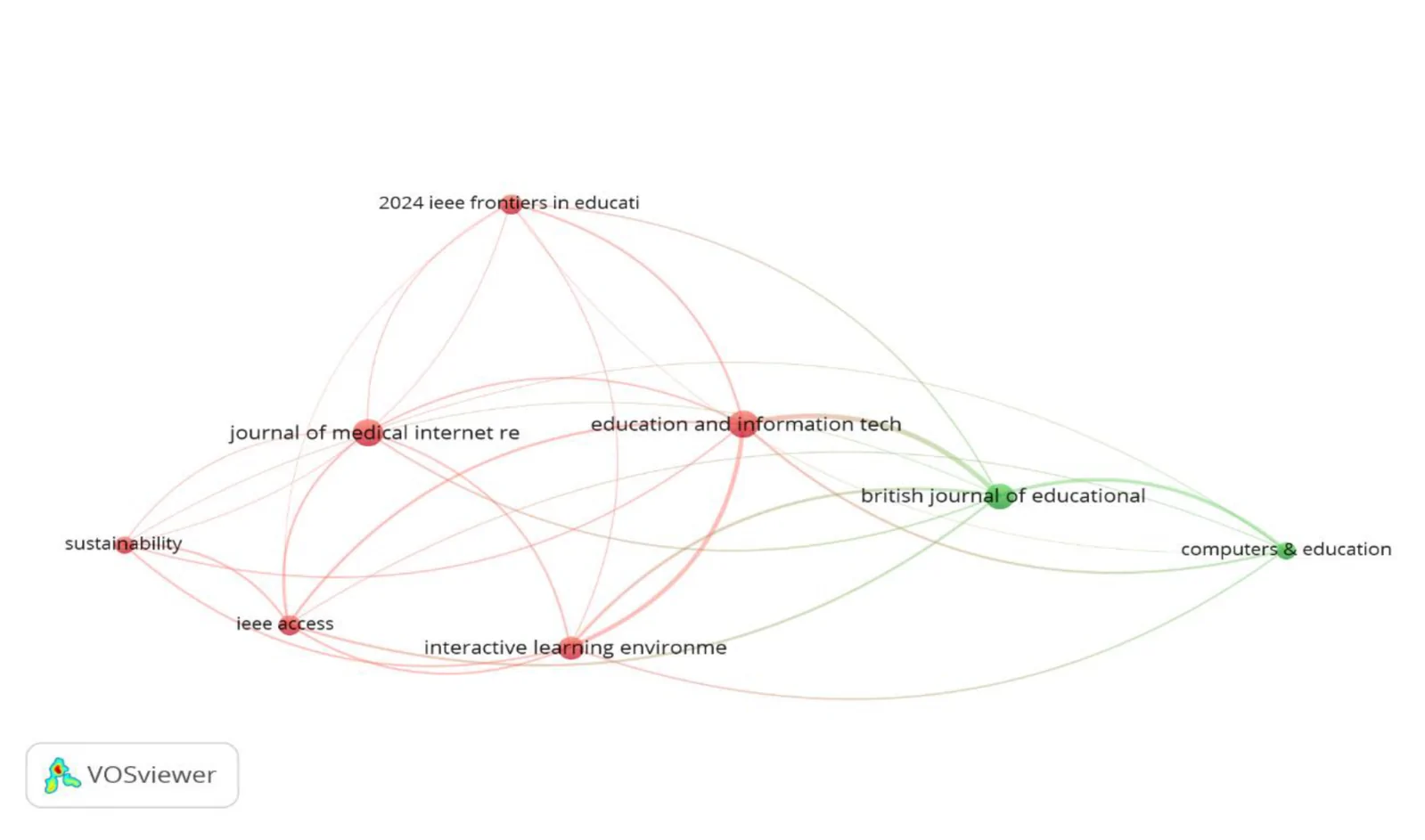
Bibliographic coupling-sources analysis based on VOSviewer network visualization.

#### 3.4.1. Red-colored cluster

In this cluster, the major literature sources are “2024 IEEE Frontiers in Education,” “Journal of Medical Internet Research,” “Sustainability,” “IEEE Access “ and “Interactive Learning Environment.”

In terms of content, this cluster mainly focuses on the intersection of technology and education, with an emphasis on medical-related educational research (Journal of Medical Internet Research), as well as broader aspects of educational technology such as interactive learning environments and IEEE-related educational frontiers. Sustainability is also a key theme, suggesting that educational research in this cluster considers both long-term and environmental impacts.

#### 3.4.2. Green-colored cluster

The major literature sources in this cluster are “Education and Information Technology,” “British Journal of Educational Technology” and “Computers & education.”

In terms of content, this cluster centers on the core relationship between education and IT. The “*British Journal of Educational Technology*” and “*Computers & education*” are well-known journals in the field, indicating that the research here delves into how IT, including agents, can be integrated into educational settings, teaching methods, and curriculum design.

The network shows a complex web of relationships among different literature sources in the AI agent-education field. The red cluster on the left seems to have a more diverse range of topics, with a stronger emphasis on emerging medical and sustainable educational applications. The green-colored cluster on the right is more focused on the traditional, yet evolving, area of educational technology. The lines connecting the different clusters and nodes indicate the co-citation relationships among these literature sources, suggesting that researchers often refer to works from medical areas when conducting studies in the AI agent-education domain, reflecting the findings in Section 3.1.

### 3.5. Bibliographic coupling-countries analysis

The network visualization, as illustrated in Figure 5, depicts the scholarly contributions of the 25 countries and regions to AI agent-based education, which are organized into 4 distinct color-coded clusters. Each cluster represents a group of nations with similar collaboration patterns and citation intensities, which reveals global research dynamics in this field.

**Figure 5.**
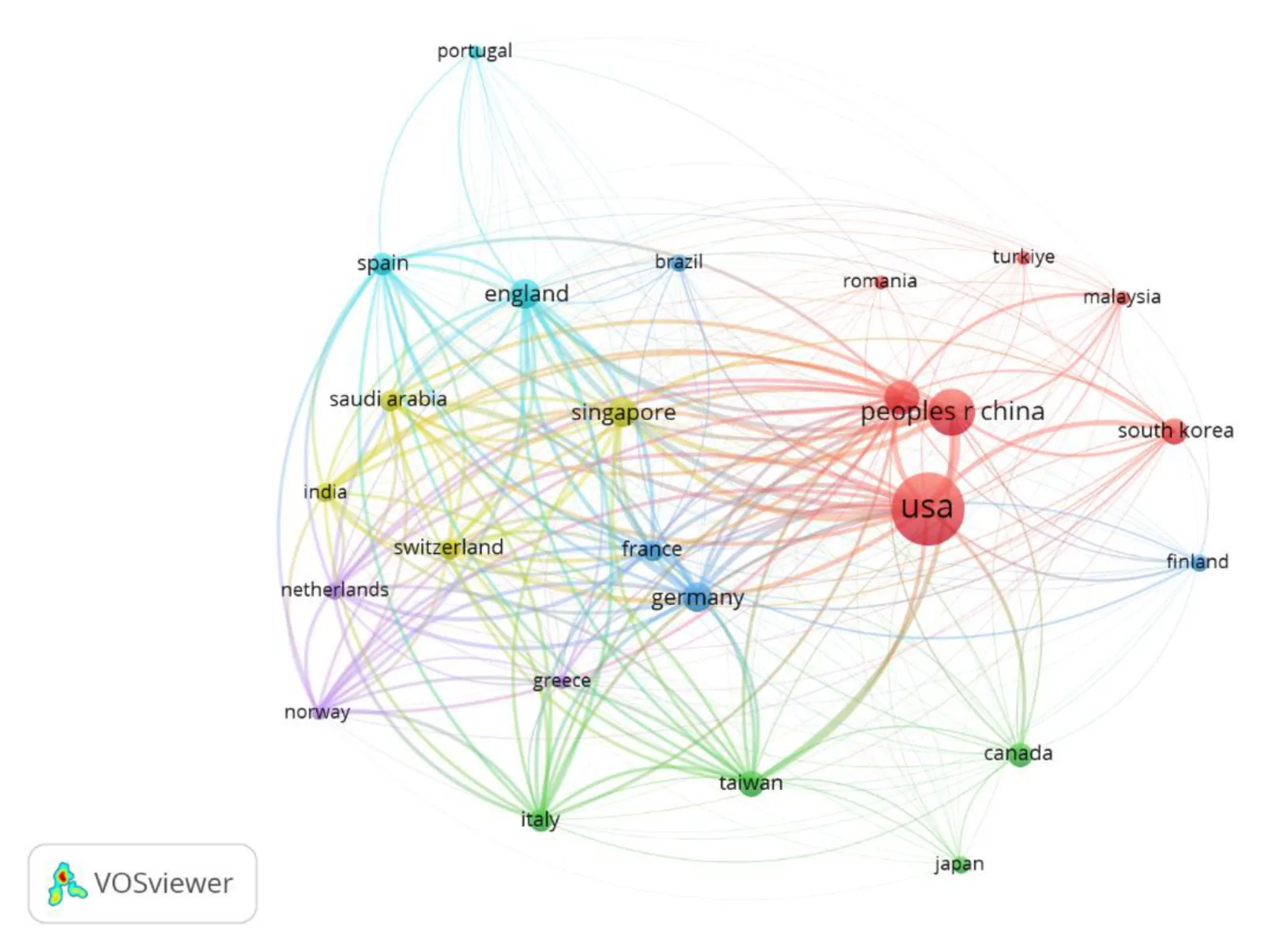
Bibliographic coupling analysis of regional contributions.

#### 3.5.1. Red cluster (high-centrality core)

The United States and China dominate the networks, with dense internal connections and numerous outbound links. The centrally located United States acts as a hub linking Europe (e.g., the UK, Germany, and France) and Asia (e.g., South Korea, Taiwan). China shows strong ties with neighboring countries (e.g., Malaysia, Singapore) and Anglophone nations (e.g., England), which reflects its growing influence on global education research. This cluster symbolizes high-impact collaborations and resource concentration in Anglo-American and East Asian academics.

#### 3.5.2. Blue cluster (European integration)

Encompassing Western and Northern Europe (e.g., the UK, Germany, France, the Netherlands, and Norway), this cluster emphasizes intra-regional collaboration. The nodes are tightly interconnected, indicating robust cross-border partnerships, particularly within the EU framework. The UK serves as a bridge to the red cluster, while Germany links to Eastern Europe (e.g., Romania), showing Europe’s coordinated efforts in educational technology research.

#### 3.5.3. Green cluster (emerging Asian hubs)

Centered around Singapore, South Korea, and Taiwan, this cluster highlights Asia’s increasing prominence. Singapore is linked to both European (UK and Switzerland) and regional partners (Malaysia and India), positioning itself as an intermediary. South Korea’s connections to China and Japan suggest intra-Asian collaboration. This cluster reflects Asia’s strategic focus on integrating AI-driven educational solutions.

#### 3.5.4. Purple cluster (peripheral collaborators)

Including Southern Europe (e.g., Spain, Portugal, Italy), Latin America (Brazil), Africa (Nigeria), and the Middle East (Saudi Arabia), this cluster features sparse connections and reliance on larger hubs. Nodes like Brazil link to Europe and North America more than to their regional peers, indicating dependency on Western academic ecosystems. Turkey’s position between Europe and Asia underscores its bridging potential, although it is underutilized in this network.

#### 3.5.5. Yellow cluster (Singapore, Malaysia, India, Saudi Arabia)

This cluster represents emerging research hubs in Asia with strong intra-regional collaborations. Singapore serves as the central node, maintaining connections with both Asian partners and Western nations such as England. This cluster reflects Asia’s growing investment in agent-based education research, particularly in multilingual and multicultural contexts.

#### 3.5.6. Teal cluster (England, Germany, France, Netherlands, Switzerland)

In a tightly knit European research network, England acts as the primary hub, linking other European countries and bridging connections to North America (United States/Canada) and Asia (Singapore). This cluster demonstrates Europe’s established research infrastructure and cross-border cooperation in educational technology, with England playing a key role in connecting continental Europe with global research networks.

## 4. Discussion and implications for future research

This section first compares this study and prior medical research, then summarizes the core findings based on the bibliometric analyses presented above and proposes implications of AI agents’ applications in education in the future.

### 4.1. Comparative analysis with prior medical research and novel contributions of this study

To situate our findings within the existing medical literature and clarify the unique contributions of this bibliometric study, we compare our results with prior systematic and scoping reviews focused on AI agents in healthcare.

Several prior reviews have made valuable contributions to understanding AI CAs in medical contexts. Følstad et al proposed an interdisciplinary research agenda for chatbots, offering a foundational framework for future inquiry. Laymouna et al conducted a rapid review of chatbots in healthcare, systematically identifying user groups, functional roles, benefits, and limitations, thereby providing a comprehensive narrative overview.^[24]^ Schachner et al rigorously reviewed AI CAs for chronic conditions, drawing attention to high heterogeneity and the pressing need for standardized evaluation. Kurniawan et al similarly focused on AI-powered chatbots for chronic illness management, highlighting critical gaps in technical documentation.

Building on these important foundations, our study introduces distinct innovations and differs from prior work in 3 key respects. First, while previous reviews have not quantitatively traced the directional flow of knowledge across domains, our citation-document analysis (Section 3.1) empirically demonstrates that educational AI agent research derives its intellectual foundations almost entirely from medical chatbot and dialogue-agent literature – a finding not previously documented in medical reviews. Second, unlike earlier studies that did not employ bibliometric network analysis to map collaboration structures, our source and country analyses (Sections 3.4 and 3.5) reveal that medical AI agent research remains concentrated within health informatics journals, with limited cross-disciplinary integration into education or computer science. Third, whereas existing reviews have not systematically identified thematic borrowings and research gaps transferred across fields, our keyword co-occurrence analysis (Section 3.2) shows that core challenges – algorithmic bias, ethical frameworks, and standardized evaluation metrics – emerged first in medical applications and are now being adopted, often uncritically, in educational contexts.

### 4.2. Key findings

#### 4.2.1. Positive impact on mental health and well-being

Social robots, such as Jibo, have proven effective in delivering positive psychology interventions, leading to measurable improvements in students’ psychological well-being, emotional states, and behavioral motivation. For instance, structured interactions with AI coaches increased participants’ happiness scores by approximately 22% and readiness to change by approximately 12%. These findings highlight AI’s role of AI in addressing mental health challenges in educational settings, particularly when combined with human-like social behaviors (e.g., expressive gestures and empathetic responses).

#### 4.2.2. Enhanced learning engagement and personalization

AI agents, particularly chatbots and intelligent tutoring systems, significantly improve student engagement by providing personalized learning experiences. Hussain et al demonstrated that AI-driven tools, such as ChatGPT and educational chatbots, enhance students’ ability to learn at their own pace, maintain motivation, and achieve better learning outcomes (e.g., 23% knowledge improvement in safety training for migrant workers). The adaptability of AI to individual learning styles and needs underscores its potential to transform traditional education models.

#### 4.2.3. Ethical and practical challenges

Despite their benefits, AI agents face critical challenges, including ethical concerns (e.g., data privacy and algorithmic bias), metacognitive laziness (overreliance on AI to reduce critical thinking), and uneven accessibility. For example, ChatGPT’s answers were only 55.6% accurate in software testing education, raising questions regarding reliability. Additionally, socioeconomic disparities affect AI adoption, as seen in studies where students from lower-resource regions struggled with AI literacy compared to their peers in high-socioeconomic status environments.

#### 4.2.4. Cross-disciplinary applications and collaboration gaps

AI agents are being integrated into diverse fields such as medical training (e.g., PROSCA for prostate cancer education), vocational safety (e.g., iSafeCom for construction workers), and chronic disease management. However, collaboration between disciplines (e.g., computer science, pedagogy, and psychology) remains fragmented. The lack of standardized evaluation metrics (e.g., only 10 of 2052 studies on chronic disease chatbots met rigorous review criteria) further limits scalable solutions.

### 4.3. Implications for future research

#### 4.3.1. Personalization and adaptive learning algorithms

Future research could prioritize AI agents capable of real-time adaptation to learners’ cognitive and emotional states. Existing research highlights the role of AI in creating adaptive learning environments that are “flexible, inclusive, personalized, engaging, and effective.^[34]^ The vision of AI agents in providing real-time, personalized feedback and acting as virtual tutors is a key opportunity for tools such as ChatGPT. Furthermore, teachers anticipate that AI will significantly accelerate personalized learning, potentially leading to more flexible school systems in which students are grouped by competency rather than age. However, the challenge of resource-intensive customization is also noted, as personalization requires significant data and fine-tuning to align with specific needs and ethical principles. The complementary role of human teachers is emphasized, with studies finding that AI serves to augment rather than replace human guidance, as seen in collaborative dance learning systems and in teachers’ perceived role as facilitators of student-AI collaboration.

#### 4.3.2. Mitigating ethical risks and bias

The ethical challenges associated with AI in education are a major theme of the literature. Multiple documents point to common risks such as hallucinations, biases in training data, jailbreaks, and anonymous data collection. A comprehensive review of AI-driven CAs explicitly analyzes ethical and regulatory challenges. Similarly, the rapid adoption of generative AI in fields such as medicine has raised urgent concerns regarding “privacy, copyright, and regulation.^[35]^ The need for trustworthy AI and considerations of how perceived trust influences user adoption behavior are also a focus of research models.^[36]^ These findings directly support the need for robust ethical frameworks and bias mitigation strategies.

#### 4.3.3. Scalability and inclusivity in low-resource settings

The need for inclusivity is implied in the call for “flexible, inclusive” learning environments. Research on children’s perceptions of AI also highlights the importance of “designing inclusive AI learning activities” that account for socioeconomic and cultural differences,^[37]^ which underscores the necessity of democratizing AI tools for underserved populations and demands innovative solutions. Offline-capable AI agents (e.g., voice-based chatbots) can bridge connectivity gaps in rural areas, while culturally localized content co-designed with local educators ensures relevance. Lightweight AI models optimized for low-end devices (e.g., smartphones) reduce infrastructure dependency, as seen in Senegal’s multilingual chatbot initiatives.

#### 4.3.4. Interdisciplinary standardization and longitudinal studies

Følstad et al explicitly proposed a research agenda aiming at fostering “cross-disciplinary collaboration.” Bridging collaboration gaps requires unified metrics (e.g., standardized metrics) to assess educational impact of AI across disciplines. Longitudinal studies tracking effects of AI over certain years are critical, for instance, evaluating how AI-assisted learning influences career readiness or lifelong learning habits. Cross-sector partnerships, comparable to open-source platforms such as GVGAI (General Video Game Artificial Intelligence), can accelerate innovation by pooling expertise from academia, industry, and policymakers. The challenges include reconciling divergent methodologies and ensuring data interoperability. A global alliance can harmonize efforts and foster scalable evidence-based AI solutions.

Taken together, future research should underscore AI’s transformative potential while highlighting the need for ethical, inclusive, and collaborative approaches to realize its full benefits in education. AI agents hold transformative potential for education, but realizing this requires addressing technical, ethical, and logistical barriers. By prioritizing adaptive design, ethical safeguards, equitable access, and collaborative rigor, future research can ensure that AI serves as a sustainable force for educational equity and excellence.

## 5. Conclusion

This bibliometric analysis systematically maps the evolution, thematic structure, and collaborative networks of research on AI agents in education. The findings reveal that the field is rapidly maturing yet remains deeply intertwined with its origins in healthcare applications. Citation-document analysis highlighted 5 clusters where AI agents enhance specialized training (e.g., safety and chronic disease management), with education emerging as a distinct domain. Co-occurrence analysis of all keywords identifies 6 interconnected networks spanning core AI technologies, human-computer interaction, educational personalization, ethical considerations, and societal impacts. Bibliographic coupling-document analysis further delineates 9 thematic clusters, underscoring AI agents’ roles in personalized learning, collaborative environments, teacher development, mental health support, and cross-disciplinary applications such as healthcare education. Bibliographic coupling-source and country analyses emphasize global collaboration, with established hubs in Europe and North America and emerging contributions from Asia, although interdisciplinary integration remains fragmented.

Therefore, this synthesis highlights AI agents’ potential to improve psychological well-being, health/medical care, engagement, and personalized learning outcomes as evidenced by studies on social robots, chatbots, and virtual tutors. However, challenges such as ethical risks (e.g., bias and data privacy), uneven accessibility, and lack of standardized evaluation metrics persist. Future research directions, as proposed, must prioritize adaptive algorithms for real-time personalization, ethical frameworks to mitigate bias, scalable solutions for low-resource settings, and longitudinal interdisciplinary studies to bridge collaboration gaps.

## Acknowledgments

We authors would like to express our sincere gratitude to Dr Binglan TU and Dr Chuanjiang Liu for their valuable guidance and critical review throughout the writing process of this manuscript. Their insightful comments and constructive suggestions have greatly improved the quality of this work. Any remaining errors are solely the responsibility of the authors.

## Author contributions

**Data curation:** Xinhuang Tang.

**Formal analysis:** Jinyan Chen.

**Funding acquisition:** Xinhuang Tang.

**Methodology:** Xinhuang Tang, Shukun Chen.

**Resources:** Xinhuang Tang, Shukun Chen.

**Software:** Xinhuang Tang.

**Supervision:** Shukun Chen.

**Writing – original draft:** Xinhuang Tang, Jinyan Chen.

**Writing – review & editing:** Jinyan Chen, Shukun Chen.
